# Response to Preoperative Therapy in Localized Pancreatic Cancer

**DOI:** 10.3389/fonc.2020.00516

**Published:** 2020-04-15

**Authors:** Giampaolo Perri, Laura R. Prakash, Matthew H. G. Katz

**Affiliations:** Department of Surgical Oncology, The University of Texas MD Anderson Cancer Center, Houston, TX, United States

**Keywords:** pancreatic cancer, preoperative therapy, tumor response, pathologic response, radiographic response

## Abstract

Evaluation of response to preoperative therapy for patients with pancreatic adenocarcinoma has been historically difficult. Therefore, preoperative regimens have generally been selected on the basis of baseline data such as radiographic stage and serum CA 19-9 level and then typically administered for a pre-specified duration as long as 6 months or more. The decision to proceed with resection following preoperative therapy likewise has rested upon the absence of disease progression rather than evidence for tumor response. This article reviews the basis for the evaluation of therapeutic response after preoperative therapy for pancreatic cancer in the existing scientific literature, and providing updates and new perspectives.

## Introduction

Pancreatic ductal adenocarcinoma (PDAC) is a extremely lethal disease, and is anticipated to be the second cause of cancer-related death in the US by 2020, surpassed only by lung cancer (1). Anatomically, localized PDAC is defined as resectable (R), borderline resectable (BR), and locally advanced (LA) based on evidence for venous and arterial involvement on cross-sectional imaging. Among all patients who present with PDAC in the USA, over 30% have a LA or BR disease and only 15% to 20% are eligible to undergo oncologic resection, the only potentially curative strategy (2).

Systemic therapy following resection improves survival outcomes relative to surgery alone (3), but with the advent of more effective chemotherapy regimens over recent years, efforts have focused on optimizing the administration of chemotherapy and/or (chemo)radiation prior to, instead of following, resection of the primary tumor. The goals of preoperative therapy are primarily to maximize the likelihood of a microscopically complete (R0) resection by reducing the size and/or anatomic extent of the tumor, to identify poor responders who progress on treatment preoperatively in order to spare them a futile operation, and to treat occult systemic disease in order to prolong survival. Practice guidelines now recognize the administration of preoperative therapy as the preferred strategy for patients with BR PDAC, while many high volume centers for pancreatic surgery are increasingly delivering it to patients with potentially R PDAC as well (4, 5).

Unfortunately, evaluation of therapeutic response to preoperative therapy has been historically difficult for pancreatic adenocarcinoma. Therefore, preoperative regimens have been typically administered, in the absence of radiographic or serologic evidence of disease progression, for a pre-specified duration as long as 6 months or more.

**Figure 1:**
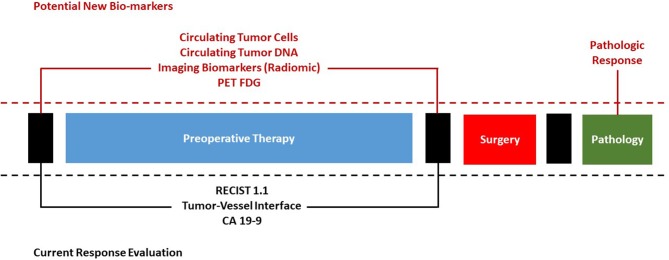
Current evaluation of response to preoperative therapy for pancreatic cancer and potential future bio-markers.

This article reviews the basis of tumor response evaluation after preoperative therapy for PDAC in the existing scientific literature and offers new perspectives to fuel the scientific debate on the important topic of surgical approach after preoperative therapy (Figure 1).

## Preoperative Therapy For PDAC

Anatomically, localized PDAC is often defined as resectable (R), borderline resectable (BR), and locally advanced (LA) according to the apparent involvement of mesenteric vasculature on cross-sectional imaging. CT and MRI studies with dedicated pancreatic-protocol are equally effective for staging, with CT being more commonly used in clinical practice (6). Vascular abutment or encasement by tumors seems to be associated with higher rates of microscopically non-radical resection, longer operative times, and higher perioperative morbidity (7, 8). Different criteria defining resectability have been proposed, all based on radiographic criteria on cross-sectional imaging. These include the expert consensus guidelines by AHPBA/SSAT/SSO/GSSC, the NCCN guidelines, and the International Study Group of Pancreatic Surgery (ISGPS) guidelines (4, 9, 10). Recognizing that patients with non-metastatic PDAC are a heterogeneous population not only anatomically, but also physiologically and oncologically, we have additionally categorized patients with PDAC on the basis of tumor anatomy (BR-A), cancer biology (BR-B), and patient comorbidities and condition (BR-C) (11).

Induction systemic chemotherapy, often followed by (chemo)radiation therapy, represents the standard of care for BR and LA PDAC, as recommended by both NCCN and ASCO guidelines (4, 5). ASCO guidelines also consider preoperative therapy as an acceptable option for patients with R disease, and specifically recommend it for patients with R tumors but radiographic findings suspicious (but not diagnostic) for extra-pancreatic disease, a performance status or comorbidity profile unfitting for a major abdominal surgery (but potentially reversible) or a CA 19-9 level (in absence of jaundice) suggestive of disseminated cancer. Today, the commonly used preoperative regimens in patients with good performance status are the combination regimens fluorouracil [5-FU], leucovorin, irinotecan, and oxaliplatin (FOLFIRINOX) and Gemcitabine-NabPaclitaxel (GA), given the efficacy of these regimens in the metastatic setting (12, 13). External beam radiation therapy with concurrent chemosensitizing 5-FU, capecitabine, or gemcitabine, or stereotactic body radiotherapy (SBRT) may also be delivered at some centers, typically following induction chemotherapy.

In the setting of LA disease, the primary goals of preoperative therapy are to reduce systemic disease burden, to reduce tumor-related symptoms, and to prolong overall survival, as only a relatively small percentage of patients with LA cancers are downstaged by preoperative therapy to an extent sufficient to make safe surgical resection realistic. The extent to which “preoperative” therapy is truly “preoperative” in such patients is therefore a matter of debate. On the other hand, the primary goal of preoperative therapy in the BR and R settings is to select patients with favorable “disease biology” who will not experience early systemic progression, as well as to improve the likelihood of complete macroscopic and microscopic resection. Together with tumor dimensions and lymph node status, in fact, R status is recognized as the most relevant determinant for prognosis in patients undergoing surgery (14–17).

The administration of preoperative therapy may also be a valuable strategy to deliver to patients with localized disease the maximum load of chemotherapy, since as high as 40% of patients will never be eligible for adjuvant treatments due to postoperative morbidity and/or failure to improve performance status following pancreatectomy (18, 19). Another purpose of primary systemic therapy is to treat occult micro-metastases at the time of diagnosis, thereby attacking the cancer foci accountable for early recurrence after resection and selecting for a “locally dominant phenotype” (20). The delivery of chemotherapy drugs prior to surgery may also allow them to better penetrate neoplastic cells since patients' tissues are still not altered by inflammation and fibrosis induced by any surgical procedure.

Preoperative therapy still presents some limitations. For example, most patients with BR PDAC have jaundice at the time of presentation. Neoadjuvant therapy necessitates the placement of biliary stents to decompress the biliary obstruction of patients with jaundice. The placement of biliary stents before surgery rises the infections risk in the perioperative period. Furthermore, many patients may require readmission or further procedures or may suffer significant complications such as pancreatitis, cholangitis and death (21, 22). Biliary stenting and preoperative therapy delay surgery, and it is not clear how the risk of progression of the disease to the point of becoming unresectable bay be increased by this delay.

No clinical trial has yet definitively clarified which therapeutic modalities are most effective as preoperative therapy for PDAC between chemotherapy, (chemo)radiation, or novel agents, and how and when each modality should be administered to maximize patient's survival and quality of life while minimizing morbidity. Therefore, current guidelines have predominantly relied upon relatively low-level data (4, 23).

Gaps in knowledge have only begun to be addressed in recent years by multicenter trials. For example, two recent trials from Korea (NCT01458717) and the Netherlands (PREOPANC-1) randomized patients with localized PDAC to either gemcitabine-based (chemo)radiation or surgery, to assess the efficacy of perioperative radiotherapy (24, 25). In both studies R0 resection rate and overall survival were more favorable in patients who received (chemo)radiation. However, although these trials suggest a possible role for (chemo)radiation in the preoperative setting, in both trial radiation was compared to surgery *de novo* and not with an alternate preoperative regimen, like systemic chemotherapy. The recently-completed Alliance for Clinical Trials in Oncology A021501 study randomized patients with BR PDAC to receive either 8 cycles of FOLFIRINOX or 7 cycles of FOLFIRINOX followed by stereotactic body radiotherapy (26). Although complete data are not yet available, it is known that the chemotherapy plus stereotactic body radiation therapy arm met the predetermined futility boundary for R0 resection and was closed prematurely. Nonetheless, local disease control or even survival for some patients with BR PDAC may still be well improved by radiation, and the role of effective local therapies can only increase if systemic therapies improve as well. After the positive results of PREOPANC-1, PREOPANC-2 (to be completed within 2022) was designed to recognize the best preoperative treatment for R and BR PDAC, randomizing patients to receive preoperative FOLFIRINOX (8 cycles) alone or preoperative gemcitabine-based chemo-radiotherapy (3 cycles) and subsequent adjuvant treatment. Finally, other phase II trials are comparing different preoperative chemotherapy regimens. SWOG S1505 for example is a randomized phase II study comparing modified FOLFIRINOX vs. GA as preoperative therapy for resectable PDAC (27). ESPAC-5F is a 4-arm phase II trial comparing upfront surgery vs. different options of preoperative strategies for patients with BR PDAC. Enrolled patients are randomized to undergo upfront resection or either preoperative gemcitabine plus capecitabine (8 weeks), preoperative FOLFIRINOX (8 weeks) or preoperative (chemo)radiation.

## Assessing Response To Preoperative Therapy

The optimal duration of preoperative treatment is not well understood. Therefore, the durations with which chemotherapy is administered in the preoperative setting have been somewhat arbitrary. While patients with progressive disease can be easily identified and spared ineffective surgery, the decision to proceed with surgical exploration following preoperative therapy usually rests on evidence of disease stability after a highly variable amount of chemotherapy with or without subsequent (chemo)radiation. We use to treat patients with BR PDAC with systemic chemotherapy for ~4 months, followed by radiation therapy, attempting to individualize patients' treatment also on the basis of clinical parameters such as their physiologic profile and serum carbohydrate antigen 19–9 level, instead of anatomy alone. However, a multimodal evaluation of the response to preoperative therapy that uses novel biomarkers and tools for response prediction is very much needed to ensure accurate selection of patients for surgery and inform treatment decisions like therapy sequencing and optimal chemotherapy regimens.

### Radiographic Response

The accuracy of radiologic assessment of response to preoperative therapy has been historically challenged. Changes in tumor size on diagnostic imaging as assessed by RECIST 1.1 (Response Evaluation Criteria in Solid Tumors version 1.1) (28), although reflective of therapeutic efficacy (or lack thereof) in other cancer treatment settings, have been felt to be insufficient in predicting response—and in particular respectability—in the setting of preoperative therapy for PDAC. The problem was identified clearly as early as 2001, with a study that suggested that CT after preoperative therapy seemed to underestimate the possibility of resecting a tumor to negative margins (29). The peculiar nature of the PDAC extensive and dense fibrous stroma, which after chemotherapy is often associated with a persistence of fibrosis tissue that prevents the tumor from shrinking on imaging—even after the destruction of cancer cells—seems to be the main responsible for this perceived lack of accuracy (30).

In 2012, for example, we reported that among 129 patients treated with preoperative gemcitabine-based therapy for BR PDAC from 2005 to 2010, only 15 (12%) experienced RECIST PR and the tumor of only 1 (0.8%) patient was downstaged to R (31). However, 85 (66%) of patients were still able to undergo resection, 81 of whom with negative margins (R0). A 2013 study showed diminished performance of CT scan accuracy predicting R0 resectability or unresectability after preoperative therapy with gemcitabine or 5-Flourouracil when compared to patients treated with first-line surgery (58% vs 83% and 52 vs 88%, respectively) (32). A study from the same year, despite being carried out in a small number of selected patients, concluded that neither changes in tumor CT attenuation nor changes in vascular involvement contributed to the prediction of resectability, and its findings were partially confirmed by a later study performed on a larger cohort, showing that only a partial decrease in tumor-vessel contact or even small decrease in tumor size was associated with R0 resection, in contrast to changes in tumor attenuation (33, 34). Focusing on 40 BR or LAPC PDAC preoperatively treated with FOLFIRINOX, a 2015 study concluded that after preoperative therapy images no longer predict unresectability, as 70% of them were re-classified as BR or LAPC after therapy although an R0 resection was achieved in 92% of them (35). Similar results were achieved in another multicenter retrospective study with 36 patients treated with FOLFIRINOX, where despite a significant tumor shrinkage after therapy, preoperative CT failed to accurately predict resectability (36).

In a recent study by Truty and colleagues, among 194 patients with BR or LAPC PDAC treated with “total neoadjuvant therapy” with FOLFIRINOX or GA, 28% had radiographic downstaging (37). A similar rate of radiographic downstaging, strikingly higher than that reported in the past, maybe due to a higher efficacy of modern chemotherapy regimens, but it may also simply reflect artifact of study conduct (such as, in this case, the exclusion of patients who did not undergo surgery). Regardless, in this study, radiological downstaging was not associated with overall and recurrence free survival.

It is interesting to notice how the majority of these studies focused on radiologic prediction of resectability, or rates of radiographic downstaging (based on vascular anatomy) in surgical series, rather than trying to assess possible radiographic predictors of tumor response to chemotherapy and survival. True response metrics would be important to determine if and how well a regimen was working preoperatively, limiting the possibility that a patient with otherwise R disease could be treated with an ineffective regimen for an inappropriate length of time. RECIST is an important, standardized and reproducible classification system, used to report and compare response rate to therapy in most prospective clinical trials, despite historically not being widely used in this clinical setting. However, it remains limited as it relies upon 2-dimensional measurement of maximum tumor diameter and uses a fixed cutoff of 30% to discriminate between a stable disease and a partial response. More objective metrics of tumor response to therapy (based, for example, on tridimensional tumor volume) may be useful to inform the delivery of induction therapy and to better assess the actual systemic response to preoperative chemotherapy, guaranteeing a more individualized approach to treatment that has higher resolution than simple dichotomous determination of resectable/not resectable.

### Serologic Response

Cancer antigen 19-9 (CA 19-9) measurement is the only biomarker for monitoring the response to therapy in PDAC approved by the US Food and Drug Administration, and it has been incorporated into the clinical staging and treatment algorithms of patients with both localized and metastatic disease. However, despite being the most commonly used marker to track response or recurrence in this setting, its use is limited to patients with a Sialyl-Lewis^A^-positive genotype (~90% of patients). Furthermore, proper interpretation of CA 19-9 measurements requires a normal bilirubin level, and elevated CA 19-9 may also be associated with inflammatory processes such as radiation therapy (38).

With the above-mentioned limitations, the change in serum CA 19-9 that happens during the administration of preoperative therapy can be clinically used as a surrogate of response to treatment and as a marker of long-term prognosis, as CA 19-9 normalization is a favorable clinical indicator associated with prolonged overall survival. In 2010, we showed how normal pretreatment and posttreatment CA 19-9 levels had high positive predictive values for respectively completing preoperative therapy and undergoing resection, despite low negative predicting values compromising their clinical utility (39). In a later study, we found that CA 19-9 normalization was associated with longer survival among both resected and non resected patients with BR disease (40).

The largest study investigating CA 19-9 as a predictor of response included 454 metastatic patients treated with gemcitabine with or without nab-paclitaxel. Ninety-six percent of radiographic responders in this study showed a decrease also in CA 19-9, compared to 78% of patients who were radiographically stable after chemotherapy, and a decrease in CA 19-9 levels predicted a survival benefit (41). The decrease of CA 19-9 after chemotherapy was also investigated as a part of the ACCORD/PRODIGE4 trial, comparing FOLFIRINOX to gemcitabine in 160 metastatic patients. Patients with a CA 19-9 ≥20% had an overall response rate significantly higher than patients with CA 19-9 <20% (44 vs. 22.9%) (42).

Recently CA19-9 normalization, rather than the magnitude of change, has been confirmed to be the strongest prognostic marker for long-term survival in a retrospective series of 131 patients with elevated (>35U/dl) CA19-9 at diagnosis who underwent preoperative therapy and resection (43). Furthermore our institution recently showed how a major pathologic response is really unlikely in patients who have elevated CA 19-9 after preoperative therapy. In this study, among 28 patients having a major pathologic response to preoperative therapy, 27 (96%) had a normal posttreatment CA 19-9 despite an elevated pretreatment CA 19-9 in 75% of the cases (see below, “Pathologic response”) (44).

The measurement of serum CA 19-9 both prior to and following the administration of preoperative therapy for PDAC is supported by these findings, and its role is emphasized as an easily assayed marker that provides insight into the biology of each patient's tumor and the patient's likely long-term outcome following completion of multimodal therapy.

### Pathologic Response

Tumor response to preoperative therapies may be measured histologically by the extent of residual viable cancer in the resected specimen. Although preoperative therapy is currently widely used, the tumor regression grading system for PDAC following preoperative therapy is not standardized. Six different systems are being used in the evaluation of PDAC following preoperative therapy, from Evans et al. to Chatterjee et al. (MD Anderson) and College of American Pathologists (CAP) 2017 (29, 45–49). Most of the systems are based on the evaluation of the destruction of viable cancer cells and/or the extent of fibrosis induced by the treatment. This metric has important prognostic implications for patients who have undergone resection of PDAC, as we previously demonstrated. Patients who experience either pathologic complete response (pCR, no viable cancer cells) or pathologic major response (pMR, <5% of residual viable cancer cells) to preoperative therapy live significantly longer than patients who have 5 to 100% viable residual cancer cells in their specimen (48, 50, 51). Unfortunately, a pCR or even a pMR in PDAC is rather rare compared to other solid tumors like breast cancer and colorectal cancer and is seen in only 3 to 11% of resected specimen treated with preoperative therapy. An accurate, standardized, and repeatable method for pathology examination of the residual cancer tissue following preoperative therapy is needed to better compare publications on this topic and to establish pathways for the diagnostic and therapeutic management of these patients.

Only few, retrospective studies have related pathologic response after preoperative therapy for PDAC to possible clinical, radiographic and serologic predictors. In 2017, a study from MD Anderson showed that baseline factors including young age, pretreatment CA 19-9 level, and use of gemcitabine as a radio-sensitizer were associated with pMR. Two other studies correlated positron emission tomography (PET) complete metabolic response and CA 19-9 response to pCR (37, 52, 53).

In a recently published study, we sought to identify potential radiographic and serologic predictors of pMR, occurring in only 28 (10%) patients, even after modern preoperative regimens. We found that posttreatment CA 19-9, RECIST Partial Response and % of tumor volume decrease were independent predictors of pMR, and a volume loss of at least 55% of the baseline tumor volume after treatment had a sensitivity of 78% and a specificity of 75% in predicting pMR (44). More importantly, pMR was extraordinarily unlikely in the absence of a posttreatment CA 19-9 within the normal range or a reduction in tumor volume with therapy. In this study, patients experiencing a pMR were confirmed to have a strikingly higher median overall survival compared to non-pMR patients (not reached vs 38 months; *P* < 0.01).

### Future Perspectives: Bio-Imaging And Bio-Markers

Novel radiomic and serologic predictors of response are actively being investigated, and it is very likely that not far in the future functional imaging or quantitative bio-imaging will provide fundamental advances.

At our institution, Amer et al. recently evaluated 4 cohorts of patients and showed that in each, the change in the radiographic interface between tumor and adjacent pancreatic parenchyma that often occurred in association with (chemo)radiation was associated with outcome. Moreover, in one of the cohorts, patients who met criteria for a radiomic response had a greater likelihood of achieving a pMR or pCR (21 vs. 0%, *P* = 0.01) (54). Our group has also identified an imaging biomarker that can be assessed using routine computer tomographic images and may be used to stratify patient's tumors into distinctive biophysical subtypes (55).

Preoperative therapy may also affect a patient's muscle mass and adipose tissue. We studied anthropometric changes occurring in patients during preoperative therapy for PDAC (56). We showed that up to 52% of patients met anthropometric criteria for sarcopenia prior to surgery, and how further depletion of skeletal muscle, as well as adipose tissue, occurred during preoperative therapy. These changes did not, however, preclude resection. Conversely, a recent multicenter study showed that adipose tissue significantly decreased during preoperative treatment, while muscle mass slightly increased (57). Resected patients experienced a higher increase in muscular tissue during preoperative treatment compared with unresected patients. Although these anthropometric measures are not direct metrics of therapeutic effect, they are important and could be supplemental measures that could potentially provide additional information to help clinicians in the re-evaluation of patients after preoperative therapy.

Systemic inflammation ratios reflect the antitumor inflammation capacity of the host and have prognostic value in patients with PDAC (58). Recently, Kawai and colleagues showed how a low lymphocyte-to-monocyte ratio after preoperative therapy is associated with poorer survival outcomes. Tumor-infiltrating lymphocytes have a crucial role in enhancement of antitumor immune response, and low lymphocyte-to-monocyte ratio may have a potential role in stratification of treatment strategies (59).

Liquid biopsy has been used in numerous recent studies to detect tumor-associated biomarkers in different extractable body fluids and is hopeful to monitor response to treatment and disease progression, and to even predict patient outcome (60). Circulating cell-free tumor DNA (ctDNA) is a tumor-associated biomarker released in the bloodstream as a result of tumor cells death and represents the molecular signature of cancer cells. Blood samples is much less invasive compared to tumor biopsies a can represent cancer heterogeneity to a larger extent. In case of chemotherapy response, therapy-induced tumor cell death should lead in theory to an increase of ctDNA levels, but in practice ctDNA levels will eventually become undetectable. Increasing ctDNA levels in the long term could indicate disease progression as a result of increasing tumor burden. Bernard et al. calculated the fraction of mutant KRAS in circulating exosomal DNA and found that an increase was associated with disease progression, suggesting how longitudinal monitoring using liquid biopsy samples through exoDNA and ctDNA provides both predictive and prognostic information relevant to therapeutic stratification (61).

Circulating tumor cells (CTCs) fluctuations were also reported to be used to monitor disease progression and clinical response to therapy in patients with PDAC (62, 63). A recent study by the Johns Hopkins University found a significantly lower number of total CTCs in patients who received preoperative chemotherapy, compared to patients who did not and preoperative number of CTCs was the only predictor of early recurrence within 12 months from surgery. In the future, CTCs dynamic may serve, at least to some degree, also as a readout of pathologic response, helping to inform the delivery of preoperative therapy and to better assess the actual systemic response to preoperative chemotherapy, guaranteeing a more individualized approach to treatment.

## Conclusion

Current radiographic and serologic evaluation of tumor response to preoperative therapy is limited for PDAC, and therapeutic decisions are typically made on the basis of absence of progression rather than evidence of tumor response. Novel biomarkers reveal the high potential for longitudinal monitoring and the use of real-time radiographic or circulating biomarkers to direct therapy. Prospective studies with a complete set of information including liquid biopsies and pathology are needed to give us a whole picture of the status of patient response to preoperative therapy. Eventually, clinical characteristics, pathology features and biomarker expression before and after preoperative therapy should be all combined to enhance the clinical significance of these biomarkers in the context of precision medicine and give a comprehensive, personalized evaluation of tumor regression.

## Author Contributions

GP devised the structure and focus of the article and wrote the first draft of it. LP wrote portions of it and edited the first draft. MK managed the manuscript through its composition, final revision and publication processes. All authors contributed to manuscript revision, read, and approved the submitted version.

### Conflict of Interest

The authors declare that the research was conducted in the absence of any commercial or financial relationships that could be construed as a potential conflict of interest.
